# The Maze of the Cerebrospinal Fluid Discovery

**DOI:** 10.1155/2013/596027

**Published:** 2013-12-12

**Authors:** Leszek Herbowski

**Affiliations:** Neurosurgery and Neurotraumatology Department, District Hospital, Arkońska 4, 71-455 Szczecin, Poland

## Abstract

The author analyzes a historical, long, and tortuous way to discover the cerebrospinal fluid. At least 35 physicians and anatomists described in the text have laid the fundamentals of recognition of this biological fluid's presence. On the basis of crucial anatomical, experimental, and clinical works there are four greatest physicians who should be considered as equal cerebrospinal fluid's discoverers: Egyptian Imhotep, Venetian Nicolo Massa, Italian Domenico Felice Cotugno, and French François Magendie.

## 1. Introduction

**Figure 1:**
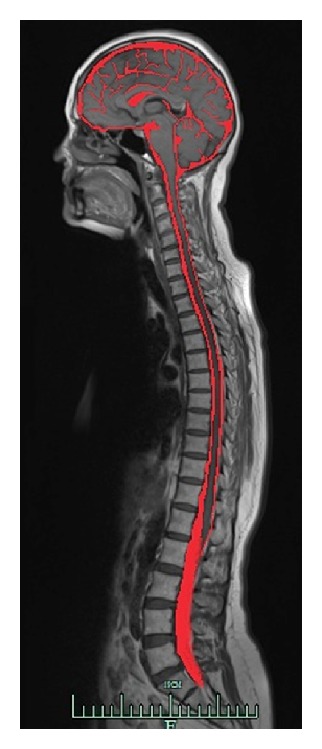
The MRI sagittal neural tube section. The cerebrospinal fluid surrounding the brain and the spine within subarachnoid space is red-colored on this image.

Cerebrospinal fluid (Latin: *liquor cerebrospinalis*) is a liquid occupying subarachnoid space (*cavum subarachnoideale*) and brain ventricles (*ventricules cerebri*) (see Figure 1). Cerebrospinal fluid was not really discovered in terms of its liquid state of matter until the early 16th century A.D. It took three more centuries for physicians to become aware of its cerebrospinal location. Previously, it was thought that cerebral ventricles contained “spiritus animalis” (spirit of the animal). According to Schaltenbrand, cerebrospinal fluid found in humans and other higher vertebrates replaced the ocean, where 3.5 billion years ago the life had begun [1, 2]. Robertson claims that cerebrospinal fluid comes from amniotic fluid by conversion of the colloidal molecules throughout the embryonic life [3]. It is known and generally accepted by medical historians that cerebrospinal fluid has been discovered by Domenico Cotugno.

## 2. “Spiritus Animalis” Idea Resonating through Centuries 

From the ancient times to the 16th century, based on the beliefs of Hippocrates from Kos (460–370 B.C.) and Claudius Galen from Pergamon (130–200 A.D.), it was thought that “pneuma psychikon” (Greek: **πνεΰμα* *ψυχικ*óv*, Latin: *spiritus animalis*) with its mental functions was located within the cerebral ventricles [4–7].

As far as Galen's role in the history of medicine is undoubted, not necessarily all the scientists analyzed his texts literally. It was Irani who referring to the research of Torack ascribed the description of the cerebrospinal fluid to Galen [8]. Torack, in turn, gave full credit to Galen for the discovery of the choroid plexus as a site of production of cerebrospinal fluid in his publication of 1982, based on *On the usefulness of parts of the body*, Galen's work translated into English in 1968 [9]. According to Conly and Ronald (although without providing the sources) it was Galen who described cerebrospinal fluid as a “vaporous humor in the ventricles that provided energy to the entire body” [10].

On the other hand, Rocca, who conducts extensive research concerning Galen's work including her own translation of the original thoughts of the Greek philosopher, does not express directly that Galen was supposed to be aware of the cerebrospinal fluid existence. Neither did he describe a fluid within the cranium [5, 11]. However, according to Rocca's own interpretation in her monogram *Galen on the brain* “it is likely that what Galen described as “residues” (**περιττώματα**) in the ventricles were traces of cerebrospinal fluid” (see page 116, footnote no. 17) [11]. Before Galen, Herophilos of Chalcedon (335–280 B.C.), regarded as the father of anatomy and known for founding a medical school in Alexandria, was the one who described all the cerebral ventricles, although there was no claim about any fluid within them [12]. On reading some anatomy textbooks written by famous physicians, it should be stated that Galen's ingenious idea of “spiritus animalis” as the key conception to understand the function of the nervous system survived through centuries—over the Dark Ages—up to the 18th century [13].

Interestingly, the cerebrospinal fluid was not even mentioned by Diocles of Carystus (4th century B.C.) while he was describing the cerebral meninges as “meninx” [14]. According to Longatti, the ancient anatomists stated that there was some “vapor” inside ventricles, which condensed into water due to temperature decrease after the death of a human [15]. Therefore, cerebrospinal fluid could not exist in living humans [15].

*A history of the Neurosurgery*, written by Greenblatt, Dagi, and Epstein, depicted Roger Frugardi, an Italian surgeon from Salerno in 1170 and his attempts in *Practica Chirurgiae* to find an examination method to check the patency of the dura mater subsequent to skull fracture [16]. The concerns involved the possibility of leaking cerebrospinal fluid or trapping air bubbles during inspiration [16]. All in all, not only did he apply a truly modern examination method but also provided the oldest known reference to cerebrospinal fluid as liquid.

During the period between the Ancient Times and the Renaissance there were no autopsies performed. The first public autopsy was undertaken by the greatest Bolognese anatomist, Mondino de Luzzi, in 1315 [17]. Only when autopsies became more popular, Andreas Vesalius, a Flemish scholar practicing in Padua, described in his phenomenal and revolutionary anatomy work *De Humani Corporis Fabrica Libri Septem* of 1543 four cerebral ventricles containing choroid plexuses [4, 18].

It is important to mention the drawings of Leonardo da Vinci from 1490, reflecting models of the cerebral ventricles, probably based on the work of Galen. There were also later drawings created between 1504 and 1507 based on the wax molding of the bovine cerebrum reflecting anatomical details quite accurately, but unfortunately they were inaccessible for centuries [6, 19, 20]. Nevertheless, cerebral ventricles were illustrated even more accurately in the work of 1523 by Berengario da Carpi called *Isagoge Breves* [6, 21]. Di Ieva and coworkers state that Berengario da Carpi (1460–1530) could observe cerebrospinal fluid but they do not indicate the appropriate description of such fluid's existence [22]. In my opinion, there is indeed an adequate fragment regarding the cerebrospinal fluid fistula in *De Fractura Calvae sive Cranei* published by Berengario in 1518 [23]. In Lind's translation we can read: “In the same small hole I kept a tent for fifty days or thereabouts because a watery discharge kept oozing out in abundance; finally, the discharge was reduced to nothing and I took out the tent, which was a cannula, and I tried to consolidate the wound” [23].

Alessandro Achillini, Mondino's successor, a great anatomist and philosopher, and a lecturer in Bologna and Padua in 1485–1512, had permission to make an autopsy [24]. Since he examined the brain ventricles as written in *Anatomical names…*, he had a big influence on medicine development especially in the field of neuroanatomy among his students [24]. But Achillini's statement that “the brain is quite dry” as in Lind's translation of Achillini's anatomy textbook *Annotationes Anatomicae* published in 1520 did not live up to his other achievements and did not prove true [25, 26].

**Figure 2:**
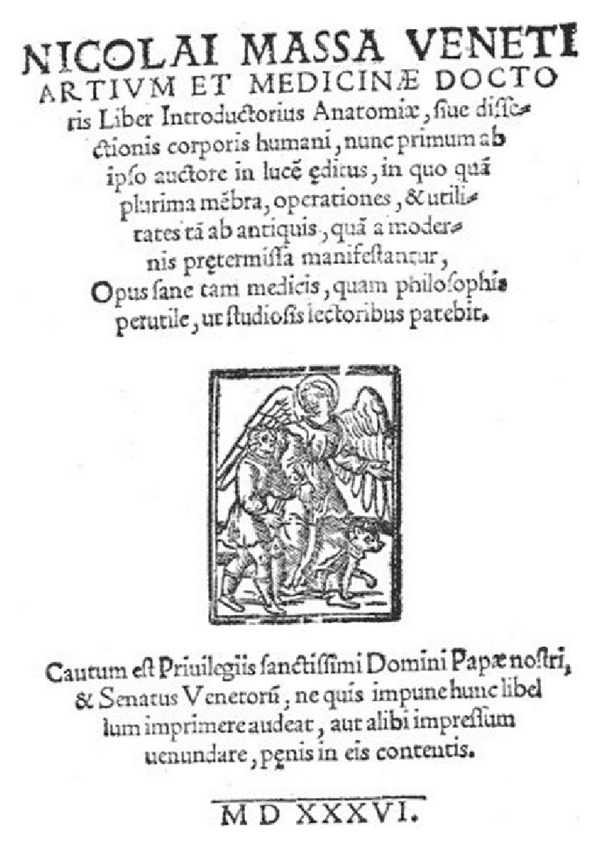
The title page of Nicolo Massa's book from 1536 (the source: e-Book Google).

Cerebrospinal fluid's research was placed on an entirely new footing eighteen years later, when a Venetian physician, Nicolo Massa, in his Latin work *Liber Introductorius Anatomiae* described a “large amount” of fluid within cerebral ventricles (see Figure 2) [27]. In his original work in chapter XXXVIII *De modo seccandi cerebri substantia ut ventriculos omnes videre possis et alias pres* on page 84 Massa wrote: “Vide […] in […] cavitatibus supfluitate aquea […] expurgate […] forame” [in English translation: see watery superfluity in ventricles escaping through foramina] and later on: “[…] semp has cavitates inveni plenas, aut semiplenas dictae aquose substantiae” [ventricles are always full or semifull of watery substance mentioned] [28]. Neither Mondino nor other anatomists during Medieval and Renaissance periods, but Massa was the first to see and report the existence of the intraventricular fluid intracranially while making an autopsy [29]. Both Wikipedia and Chudleri declare this event as a significant milestone throughout the history of neuroanatomy and neurophysiology [30, 31]. Among all of discoverers, Nicolo Massa is regarded as the first person who described cerebrospinal fluid properly.

Also representatives of the Oxford school played important role in the discovery of the cerebrospinal fluid. In 1664 Thomas Willis wrote *Cerebri Anatome*, which described the presence of fluid in the ventricles and cerebral aqueduct. He rightfully thought about active production of cerebrospinal fluid by choroid plexus but simultaneously incorrectly assumed the possibility of active intranasal drainage of the cerebrospinal fluid [9, 32]. On the other hand, Richard Lower in his pivotal discovery (described in *Catarrhis*) explained that cerebrospinal fluid did not drain into nasal cavities and that the nasal passages had nothing to do with the circulation of the cerebrospinal fluid [33]. His research involved use of contrast material (milk or other, black substance) at the base of the skull and monitoring the distribution of the contrast with special attention to the nasal cavities and throat [34].

Marcello Malpighi the first histologist, described in his treatise *De cerebro* published in 1665 that white substance of the brain was composed of the same fibers as nerves; the fibers were filled with a liquid and spread along nerves: “Cerebro humor in nervos propagetur” (p. 24 of the original Latin book) [35]. According to Malpighi the fluid was secreted by the cortical glands of the grey matter. Importantly, Malpighi never used in his book such terms as “cerebral fluid,” “nervous fluid,” “ventricular aqua,” or “liquid”. He referred all the time to Galen's “spiritus animalis” theory. He stated in 1665 that “the finest serum of this blood is filtrated through the exterior part and, then entering into the fibres of the brain, is thence conveyed into the nerves, which he affirmed to be the reason that the head is so often found full of water when the brain has received a wound or an alteration by some distemper” [36]. The editors of *Philosophical transactions* reported in footnote in 1809 that “this idea of the nerves being filled with serum, and thence producing hydrocephalus, is extremely erroneous”.

In 1683 Bellini described a fluid flow through the nerves “[…] ad motum liquidi per Nervos” and then out of the brain to the spinal cord “autem cerebrum & spinalem medullam” [37]. Work by Klass shows that Bellini and Willis must have been keenly aware of a fluid's existence at the base of the brain [38].

In a description of the brain anatomy with precise pictures of brain structures published in 1684 Raymundi Vieussens, French anatomist, referred to Galen's theory on 35 pages. He gave careful consideration to “spiritus animalis” in separate chapters (no. XV on pages 94–97, no. XVIII on pages 112-113, no. XIX pp. 113–119, and no. XX pp. 119–124), where he attributed all body movements to this phenomenon [39]. According to Vieussens “spiritus animalis” is produced in brain's cortex: “Spiritum omnem animalem in cortice cerebri stricte sumpti productum” [39]. In the first manuscript about brain anatomy in English (1695) Humphrey Ridley described the presence of cerebrospinal fluid in the ventricle system: “Aquae in quartum ventriculum” (see p. 131) [40].*‬‬‬*

Giorgio Baglivi, one of the famous physicians in the 18th century, was not only discoverer of fibrillar theory but according to Zurak also a pioneer in cerebrospinal fluid/blood barrier conception [41]. In my opinion, he also presented the concept of cerebrospinal production/absorption homeostasis in 1703: “[…] sanguis a corde ad cerebrum, et a cerebro ad cor fluidum nerveum cum aequlibrio impellantur” (see p. 66 of his book) and was aware of cerebrospinal fluid circulation: “[…] liquida per cerebrum circulantia” (see p. 69 of his *Tractatus*) [42].

Olry reports that Antonio Pacchioni, Malpighi's successor and discoverer of arachnoid granulations in 1705, stated in his previous work that these granulations were “to secrete the cerebrospinal fluid,” in the original: “[…] a sanguine derivatum fluidum” [43, 44]. Again, the mystical and incomprehensible Galen's conception won over rational understanding of body function [45]. In his works, Pacchioni pointed to cerebrospinal fluid's existence indirectly using the following terms: “liquoris guttalas,” “lympha,” “cerebrum lympha,” “humor, seu lympha,” “fluidum,” “liquor,” “fluidum nervorum,” “aquae vitae,” and “aqua communi” [44].

## 3. Shift towards Awareness of Fluid's Presence

Vesalius, Varolio, Glisson, and Haller were all aware of the fact that there was no “pneuma psychicon” within cerebral ventricles but rather cerebrospinal fluid [7]. In 1692, Antonio Mario Valsalva described the presence of cerebrospinal fluid within subarachnoid space surrounding spinal cord, while performing a canine spinal cord cross section [7, 16]. Over half a century later, in 1747, Albrecht von Haller described fluid within cerebral ventricles and on the surface of the hemispheres. He also revealed his concept of cerebrospinal fluid circulation as fluid secreted by the arteries and reabsorbed into venous system [7, 9, 16, 32]. Moreover, he was the first to describe physicochemical characteristics of cerebrospinal fluid such as its viscosity or coagulation under the influence of alcohol, strong acids, and high temperatures of 150°C [7, 9, 16, 32]. All of the above can be found in chapter XIX pp.43–46, *Aqua ventriculorum*, from the Latin edition of Haller's 1762 *Elementa Physiologiae Corporis Humani* [46]. It was in 1738 that Fantoni first explained the proper location of the cerebrospinal fluid absorption in the venous sinuses of the intracranial cavities [43].

Between 1741 and 1744 Emanuel Swedenborg, a Swedish engineer and visionary, revealed a detailed description of cerebrospinal fluid in manuscript that was not published until 1887. The document itself allegedly did not have a major influence on the development of the neuroanatomy or neurophysiology, as the Swedenborg's biographers claim [4, 32, 47]. On the contrary to their statement, just in 1764, von Haller on page 28 of his Latin anatomy and physiology textbook described blood supply of cerebral cortex referring to Swedenborg's work and thus confirming that Swedenborg's discovery was known at that time [46]. Interestingly enough, Tafel apparently treats Swedenborg as the cerebrospinal fluid's discoverer stating that Cotugno (in 1764), Magendie (in 1815), and Key and Retzius (in 1875) independently “rediscovered” cerebrospinal fluid [48].

**Figure 3:**
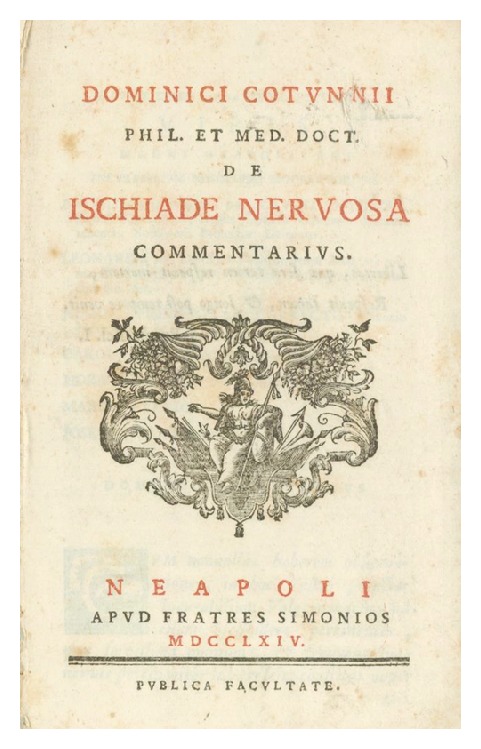
The title page of Dominici Cotunii's book from 1764 (Courtesy of Posner Memorial Collections, Carnegie Mellon University Libraries, Pittsburgh, PA).

It was the Italian physician, Domenico Felice Cotugno, who discovered and described cerebrospinal fluid in a Latin publication *De Ischiade Nervosa Commentarius* in 1764 (see Figure 3) [7, 49]. Longatii states that Contugno's discovery while researching spinal nerves of ischiadic nerve was totally coincidental [15]. In chapter IX-XV on pages 11–19 Cotugno described presence of the cerebrospinal fluid under the dura mater, within ventricular chambers, surrounding spinal cord and cerebral hemispheres [49]. Cotugno also clarified the cerebrospinal fluid circulation from the ventricles through aqueduct into subarachnoid space surrounding the brain and spinal cord. Additionally, Cotugno concluded that his discovery was possible only due to the fact that the new method of an autopsy had been introduced—without separating the head from the rest of the body. It was Cotugno's student, Crantz, who noted that his anatomy teacher's discovery was possible based on 20 autopsies performed on human bodies positioned even vertically upside down [50].

Cotugno's subsequent research revealed that cerebrospinal fluid was clear and its amount was an equivalent of the three Neapolitan ounces (1 Neapolitan ounce = 26.72 gram), which was described on page 18 in *De Ischiade Nervosa Commentarius* [4, 49]. Apparently, the cerebrospinal fluid was named for Cotugno—“Cotugno's fluid” or in Latin *liquor Cotunnii* [51]. In spite of this, Cotugno himself gave full credit for the cerebrospinal fluid discovery to Haller [50]. In chapter XIV (page 17) Cotugno underlined that beyond doubt it had been Haller who first described intraventricular fluid (long before Cotugno's rediscovery):

“Nullu attamen dubito; quo nuperrime acredidit Vir summus Hallerus (q); his spinae aquis eas etamisubindecomisceri, quas sivea majoribus cerebri ventriculis”, where the reference “q” stands for Haller's work *Elementa Physiologiae* Volume IV, Lib. X, Sect. II, p. m. 77,78 [49]. In turn, von Haller in 1762 in his work (page 43), while describing intraventricular fluid in his own way, referred to the earlier research of Massa (dating back to the 16th century) [46]. Such cases exemplify in an unequalled way that the researchers were clearly aware of their predecessors' achievements and fully recognized them treating their own work just as contribution.

It is important to note that Cotugno also confirmed the presence of cerebrospinal fluid in animals—fish and turtles—in 1784 [52]. Not only did he discover presence of cerebrospinal fluid in the subarachnoid space around the spinal cord and in the ventricles but also established the direction of the cerebrospinal fluid flow. Cotugno's work is discussed in detail by Di Ieva and Yaşargil based on its English translation of 1775 *Treatise on the nervous sciatica, or nervous hip gout* [51, 53]. It is worth mentioning that they consider both “Crantz HJN” and “Cotunnius D” to be the authors of the work, although only the latter name appears in the English version [51, 53].

According to Pearce, Domenico Cotugno was supposed to describe cerebrospinal fluid not in 1764 but in 1761 in his doctor's thesis *De aquaeductibus Auris Humanae Internae* [54]. Pearce also stated in his 2003 publication *Fragments of Neurological History* that Cotugno in *De aquaeductibus auris humanae* of 1761 presented and described “a clear watery fluid in semicircular canals of the inner ear which he observed resembling pericardial fluid and C.S.F. in the ventricles” [4]. As it turned out later on, during detailed analysis of the Vienna's version of Cotugno's doctoral thesis—on the contrary to Pearce's statement—Cotugno did not describe the existence of cerebrospinal fluid but labyrinthine one [55].

Marshall and Magoun claim that Cotugno described cerebrospinal fluid in the subarachnoid space surrounding spinal cord in *De ischiade Nervosa Commentarius* [48]. Dechef says that the initial discovery of the cerebrospinal fluid did not draw a lot of attention of researchers at that time, because it was described in the book about sciatica and not about cerebrospinal fluid itself [56]. Böni and associates consider Cotugno the first one to describe cerebrospinal fluid and its circulation [57]. At the same time they claim that 70 years before Cotugno, it might have been Valsava who mentioned the presence of cerebrospinal fluid location and circulation but they are unable to provide the source of this information [57].

In the 18th century the theory of contractile activity of the dura mater became dominant at the cost of Galen's “spiritus animalis” theory. Although Macbride rejected the idea declaring “the dura mater to be the first mover of the nervous, as the heart is of the vascular system”, he reported in his English textbook of 1772 the existence of “nervous fluid” [58]. He was also aware of fluid's secretion in the brain: “Notwithstanding the impossibility of demonstrating a nervous fluid […] but a secretion is carried on there” [58].

**Figure 4:**
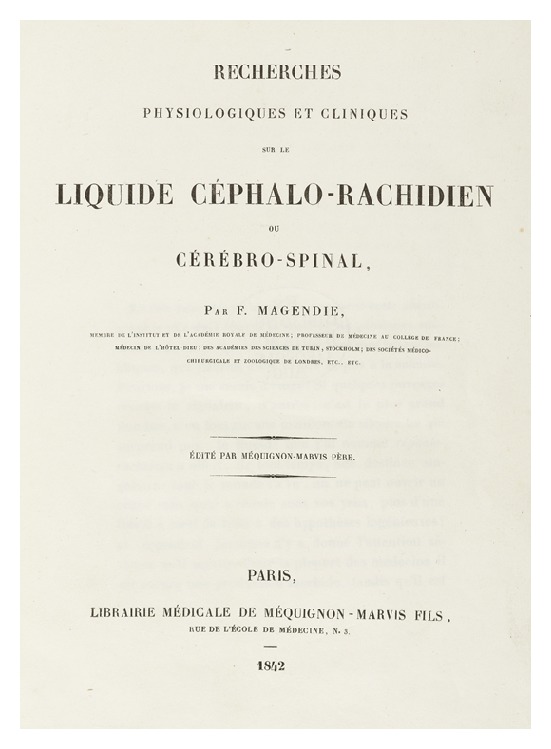
The title page of François Magendie's book from 1842 (the original book: Göttingen state and University Library).

Greenblatt, *Dagi*, and* Epstein* as well as Sato agree that it was not until 1828 that French physician, François Magendie, after describing Cotugno's works in Latin, confirmed presence of connection between cerebral ventricles and subarachnoid space, as well as continuity of these spaces around the brain and spinal cord [16, 59]. Apparently, from that moment on cerebrospinal fluid circulation has become generally accepted knowledge. Having in mind the fact that Magendie was able to prove it in an experimental research rather than postmortem studies as it was in the case of Cotugno, Magendie claimed himself as an author of the cerebrospinal fluid discovery [9]. As the following extract attests Magendie coined the term “cerebrospinal fluid” in his 1842 publication: “Recherches physiologiques et cliniques sur le liquide cephalo-rachiden ou cerebrospinal” (see Figure 4) [18]. It was also Magendie who properly described the direction of cerebrospinal fluid flow, especially the exit of cerebrospinal fluid from the fourth ventricle out of the brain. What had been the most common idea before was Bichat's assumption about cerebrospinal fluid release into the available space within the structure of a local production [60]. Last but not least, Magendie successfully introduced suboccipital puncture and method of measurement of cerebrospinal fluid pressure [61]. It is worth putting strong emphasis on crucial importance of Magendie's achievements for cerebrospinal fluid's understanding.

Research of Faivre and Luschka in 1853 allowed forming the hypothesis that cerebrospinal fluid was produced by the choroid plexus within the cerebral ventricles [62]. Another groundbreaking idea was presented by Weed in 1868. He described cerebrospinal fluid flow from cerebral ventricles through subarachnoid space to the venous sinuses of the brain [62].

Within the next few decades the final proofs of cerebrospinal fluid's properties were provided. In 1876 Key and Retzius, while performing postmortem research, proved that cerebrospinal fluid was absorbed through subarachnoid granulations into venous sinuses of the brain [63]. For the first time cerebrospinal fluid sample was obtained *in vivo* by the lumbar puncture from a patient with central nervous system tuberculosis by the physician Walter Essen Wynter in 1889 in London [64]. Two years later, the technical aspects of the lumbar tap were released to the public by a German physician, Quincke [64]. It might have been Quincke who formulated a thesis about the direction of cerebrospinal fluid circulation from inside ventricles to the surface of the brain [1]. Specific details of cerebrospinal fluid circulation were announced by Weed in 1925 and were well accepted by the medical community [65]. Weed claimed in his original work that cerebrospinal fluid being produced by the choroid plexus gets immediately into the ventricular chambers [66].

Based on all the discoveries above, in 1925, Harvey Cushing, an American neurosurgeon, treated as the father of neurosurgery, considered cerebrospinal fluid path as the third circulatory system, in addition to vascular and lymphatic ones [67]. In 2006 Madsen reported the fourth circulation system linked to cerebrospinal fluid as fluid pulsation waves going through central nervous system [68]. Kothari and Goel introduced term “neuraqua,” which is to reflect the function of the said fluid [69]. In turn, Gardner claimed that subarachnoid space with cerebrospinal fluid inside it is considered a phylogenetic artifact [70].

## 4. An Egyptian Trace

It might look like the end of cerebrospinal fluid's discoveries but there is also another interesting trace worth mentioning. In 1862, Edwin Smith, Egyptologist, discovered a fascinating piece of papyrus, translated into English by Breasted in 1930 [48, 71]. Apparently the said document had 48 clinical cases describing disorders of nervous system, bones, and joints.

It is believed that it was the outstanding physician and the architect of the ancient Egypt, Imhotep, who was the author of the papyrus dating from 3000 B.C. [71]. *The Papyrus of Smith*, dated 1600 B.C., contains a reprint from the Imhotep's original of 3000 B.C. *The Papyrus of Smith* was considered the oldest medical source of information in the area of not only diagnosis and treatment of head and spine injuries as well as sciatic nerve disorders, but also anatomic discoveries as in the case of brain or meninges [27, 48, 72].

**Figure 5:**
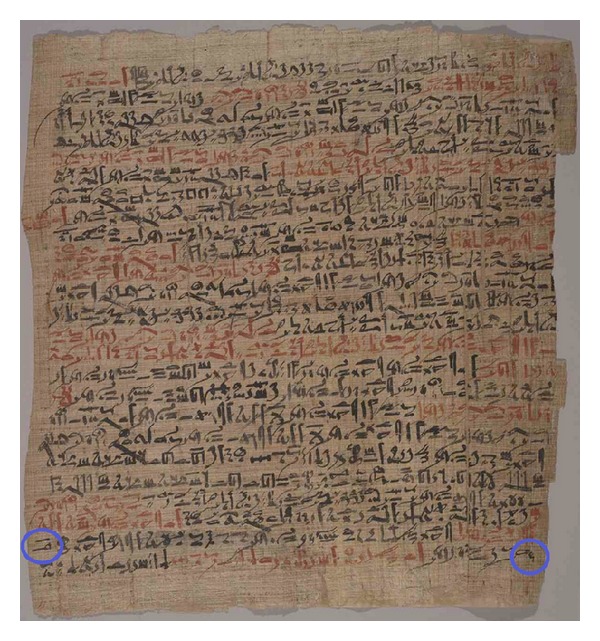
At the bottom of the 2nd column of the Edwin Smith's Papyrus, at the end of 24th line, and at the beginning of 25th one reading from left to right, we can see hieroglyphic signs (circled in blue) referring to cerebrospinal fluid (printed with permission of the New York Academy of Medicine).

In the case number 6 (see Figure 5) on pages 167 and 171, Breasted described open head trauma with skull fracture and meningeal rupture: “[…] it breaks open his fluid in the interior of his head” [73]. What is more striking is the text on page 168, where Breasted also translated that fingers of the surgeon, attending the trauma, had felt pulsation of the brain tissue similar to the heart pulsation [73].

Clark and O'Malley report that history of cerebrospinal fluid is the longest one since it was mentioned already in *The Papyrus of Smith* [74]. According to Wilkins, the document concerned is the first written acknowledgement of intracranial fluid presence [75]. It seems to be the sufficient proof of scientific discovery because the following conditions have been met: the phenomenon described in *The Papyrus* is real and actual, it was noticed directly by a careful observer, and was also made known to the public [76]. Due to the fact that a scientific discovery is not subject to the intellectual property law, there are enough necessary prerequisites to giving full credit to Imhotep for discovery of cerebrospinal fluid. Taking into consideration the content of *The Papyrus of Smith*, Imhotep can be fairly believed to be the very first discoverer of cerebrospinal fluid.

It has been pointed out that even Homer in his epic poem *The Odyssey* appreciated the outstanding medical knowledge and skills of the Egyptians in the book IV: “[…] Egypt […] Moreover, everyone in the whole country is a skilled physician” [77]. Shehata concluded in his historical consideration that “the main scientific knowledge of the Egyptian medical papyri was exactly Copie by the Greek scientists Aristo, Hippocrates, Dioscorides and Galen” in the range of anatomy, medical diagnosis, treatment, head injuries, fractures, theory of diseases, and ethical code [78]. And what is more, he also stated that he accepted Imhotep as the father of medicine [78].

## 5. Conclusions

1. The Egyptian physician Imhotep is the most likely to be the first one to discover intracranial cerebrospinal fluid *in vivo* in 3000 B.C. The description of the discovery was found in *The Papyrus of Smith* of 1600 B.C.
2. Nicolo Massa in his 1536 publication *Liber Introductorius Anatomiae* was the first one to describe cerebrospinal fluid within cerebral ventricles based on the postmortem autopsies.
3. There is historical and consensual agreement that Domenico Cotugno was the first one to discover cerebrospinal fluid through the experimental postmortem research during the autopsies. Cotugno in his *De Ischiade Nervosa Commentarius* of 1764 was the first one to describe cerebrospinal fluid in the subarachnoid space around spinal cord.
4. The name “cerebrospinal fluid” was introduced by François Magendie in the first half of the 19th century. Magendie was the first to discover method of cerebrospinal fluid pressure measurement and he was able to lay the scientific foundation for development of the cerebrospinal fluid dynamic research.
